# EXPRESSION OF CONCERN: Post‐Burn Scar Malignancy: 5‐Year Management Review and Experience

**DOI:** 10.1111/iwj.70836

**Published:** 2026-01-26

EXPRESSION OF CONCERN: A. K. Mousa, A. A. Elshenawy, S. M. Maklad, S. M. M. Bebars, H. A. Burezq, S. E. Sayed, “Post‐Burn Scar Malignancy: 5‐Year Management Review and Experience,” *International Wound Journal* 19, no. 4 (2022): 895–909, https://doi.org/10.1111/iwj.13690.

This Expression of Concern is for the above article, published online on 14 August 2023 in Wiley Online Library (wileyonlinelibrary.com), and has been issued by agreement between the journal Editor‐in‐Chief, Professor Keith Harding; and John Wiley & Sons Ltd. An investigation by the publisher found that images in Figures 8B and 8C had been duplicated and rotated, albeit with different staining. In addition, the investigation found that information regarding the study's ethical approval and informed consent procedure were unclear. In addition, the study reported in the article is not registered in a clinical trial registry, as required by the journal's guidelines.

The authors responded to an inquiry by the publisher stating that the article received approval from the Aswan University ethical committee and that the study was not registered in a clinical trial registry because no trial management protocol was applied to the study. The authors did not provide original ethical approval documents as requested and did not provide an explanation for the duplicated images nor all original data for the study. The Expression of Concern has been agreed to because the concerns regarding a duplicated image and lack of clarity regarding the ethics and informed consent procedures remain unresolved. The authors were informed of this Expression of Concern.

